# Candida Esophagitis in Patients with Solid Organ Cancers

**DOI:** 10.3390/jcm15041474

**Published:** 2026-02-13

**Authors:** Ahmed Telbany, Hannah Farfour, Krista Gomez, Youssef Soliman, Toufic A. Kachaamy

**Affiliations:** 1 Department of Gastroenterology, University of New Mexico, Albuquerque, NM 87106, USA; 2 Edson College of Nursing and Health Innovation, Arizona State University, Phoenix, AZ 85004, USA; 3 Department of Gastroenterology, City of Hope Phoenix, Goodyear, AZ 85338, USA

**Keywords:** Candida esophagitis, esophageal candidiasis, solid tumor, immunosuppression, antifungal therapy, opportunistic infection

## Abstract

Candida esophagitis (CE) is the most common fungal infection of the esophagus and an increasingly recognized complication in patients with solid organ malignancies. Once primarily associated with advanced HIV/AIDS and hematologic malignancies, the epidemiology has shifted in the modern era of antiretroviral therapy and intensive cancer treatments. Patients with solid tumors receiving chemotherapy, corticosteroids, broad-spectrum antibiotics, and proton pump inhibitors (PPIs) are at a heightened risk for CE due to synergistic immunosuppressive and mucosal barrier-disrupting effects. Clinically, CE in cancer patients often present with odynophagia, dysphagia, or chest pain, but a considerable proportion of cases are asymptomatic or non-specific, complicating diagnosis and needing a high index of suspicion. Endoscopic evaluation with characteristic white plaques and histopathologic confirmation remains the diagnostic gold standard, as symptoms as oropharyngeal findings are unreliable indicators of esophageal infection. Disease management centers on systemic antifungal therapy. Fluconazole is the first-line treatment, achieving high cure rates, while echinocandins and posaconazole are reserved for refractory cases or non-*albicans* infections. Prompt therapy is crucial, as untreated CE can lead to malnutrition, interruptions in cancer therapy, and rare but serious complications (e.g., necrotizing esophagitis or perforation). We provide a comprehensive review of the epidemiology, risk factors, clinical manifestations, pathogenesis, diagnosis, and management of CE in solid organ cancer patients. Gaps in knowledge are highlighted, including the need for better non-invasive diagnostics, antifungal resistance surveillance, and tailored prophylactic strategies. A high index of suspicion and early recognition and treatment of CE in oncology patients can improve nutritional status, quality of life, and continuity of cancer care.

## 1. Case Vignette

**Figure 1:**
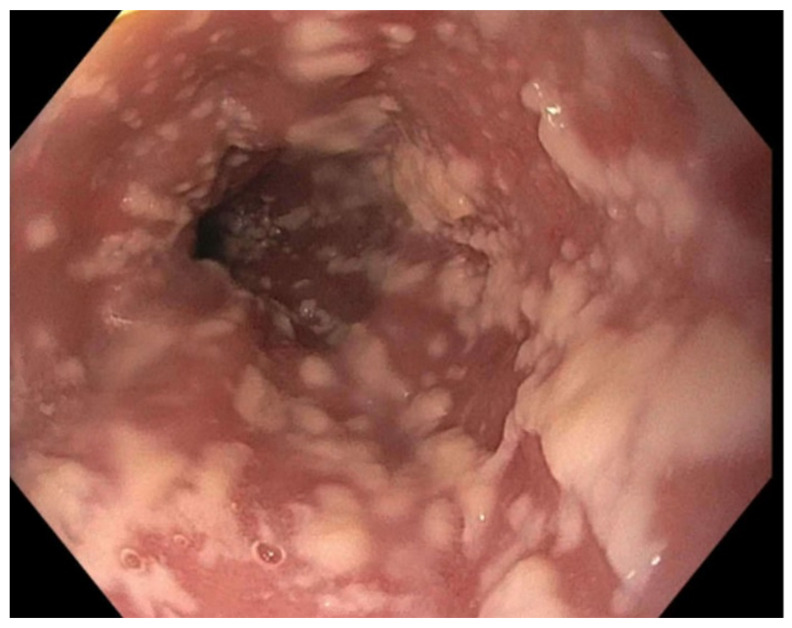
Endoscopic appearance of Candida esophagitis with white cotton-like plaques.

**Figure 2:**
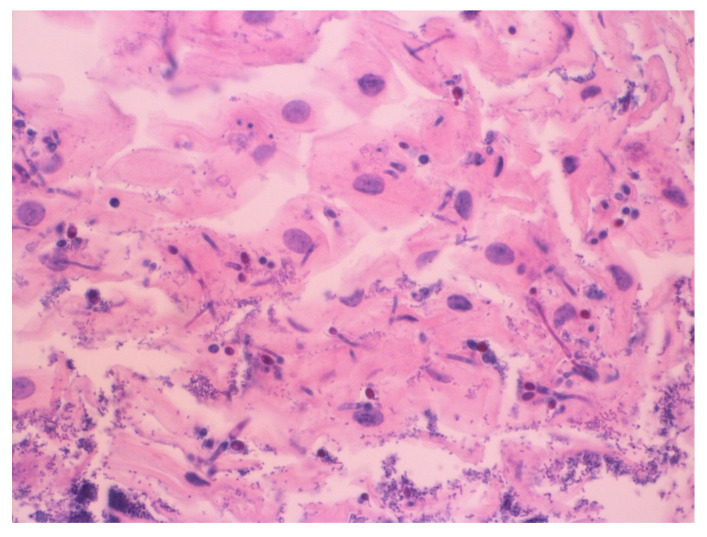
Candida esophagitis under hematoxylin and eosin (H&E) stain shows fungal organisms, specifically branching pseudohyphae and budding yeast, invading the superficial squamous epithelium.

**Figure 3:**
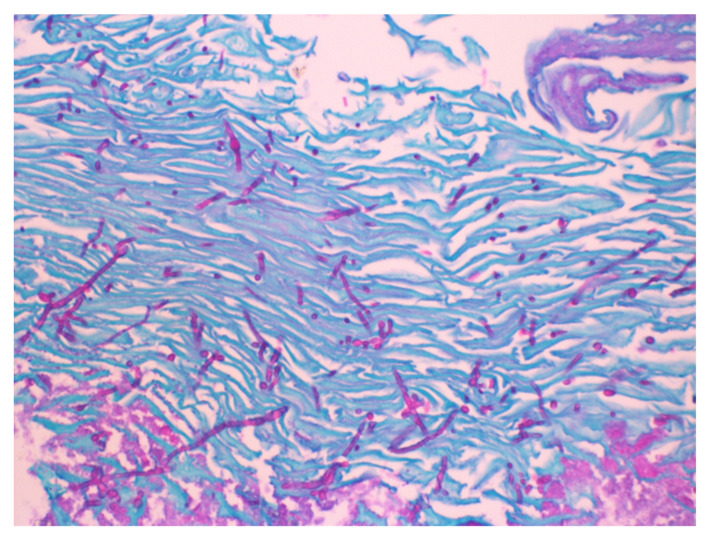
Periodic acid–Schiff (PAS) highlights the fungal elements against the surrounding tissue, with the fungal cell walls of Candida shown in magenta.

A 77-year-old man with stage IV pancreatic adenocarcinoma on his fifth cycle of FOLFIRINOX chemotherapy presented with intractable upper abdominal pain. The pain was constant in the epigastrium, radiating to the back, and worsened by oral intake. He had unintentionally lost over 9 kg in 3 months. High-dose opioid analgesics provided partial relief but caused constipation, nausea, and early satiety. On examination, he appeared cachectic with mild epigastric tenderness; the oral mucosa was normal. An endoscopic ultrasound-guided celiac plexus neurolysis was planned for pain control. During the procedure, diffuse white plaques were incidentally noted lining the esophageal mucosa (Figure 1). Biopsy and cytology confirmed Candida esophagitis (Figure 2 and Figure 3). Notably, the patient denied odynophagia, dysphagia, or heartburn despite these findings. He started on oral fluconazole, and, at follow-up, his esophageal candidiasis had been resolved without further interrupting cancer therapy.

## 2. Introduction

Esophageal candidiasis, or Candida esophagitis (CE), is the most frequent fungal infection of the esophagus and a significant opportunistic infection in immunocompromised hosts. It has long been recognized in patients with advanced HIV/AIDS and hematologic malignancies; however, over the past two decades, a marked epidemiologic shift has occurred, with rising incidence among patients with solid organ cancers. This trend parallels broader oncology demographics: solid tumors account for over 90% of all cancers worldwide, and advances in cancer therapies have extended patient survival while often inducing susceptibility to opportunistic infections [1]. Consequently, CE has emerged as an underappreciated complication in solid tumor populations, with few studies or publications specifically on this topic [2]. We performed a comprehensive literature search of the PubMed, Embase, and Google Scholar databases for English-language articles on ‘Candida esophagitis’ or ‘esophageal candidiasis’ in solid tumor patients, published from 2000 through 2025. We manually reviewed the references of relevant papers to include additional pertinent studies to write this narrative review on this topic.

*Candida* species, particularly *Candida albicans*, are commensals of the human oropharynx and gastrointestinal tract. In healthy individuals, intact mucosal immunity and microbial balance prevent Candida overgrowth. In the setting of immune suppression or mucosal injury, however, these fungi can convert to a pathogenic state, adhering to the epithelium and invading superficial mucosa [3,4]. Among patients with solid organ cancers, multiple factors create an ideal milieu for Candida proliferation and esophageal colonization: nutritional deficiencies, cytotoxic chemotherapy, and radiotherapy damage the mucosa; corticosteroids impair cell-mediated immunity; broad-spectrum antibiotics disrupt normal flora; and proton pump inhibitors (PPIs) reduce gastric acidity. In addition, esophageal diseases such as esophageal cancer or alterations in the esophageal anatomy, such as those caused by esophageal surgery, likely interfere with esophageal function, further favoring Candida overgrowth [2,5,6,7,8]. These iatrogenic and disease-related factors distinguish solid tumor patients from those with hematologic malignancies, where profound neutropenia and stem cell transplantation are the dominant risk factors for invasive fungal infections [2,9].

Clinical manifestations of CE range from the classic symptoms of odynophagia (painful swallowing) to dysphagia (difficulty swallowing), retrosternal chest pain, nausea, and epigastric discomfort. Importantly, a considerable proportion of patients, especially in solid tumor populations, may have minimal or no esophageal symptoms [2,10]. Oropharyngeal thrush is an unreliable predictor of esophageal involvement and is less common than previously thought, reported to be as low as 2% in some series [2]. The consequences of missed or delayed diagnosis of CE in cancer patients are significant. Untreated infection can lead to progressive poor oral intake, weight loss, and interruption of cancer therapies. A high index of suspicion and the timely recognition and treatment of CE are therefore critical to optimize oncology patient outcomes.

Given the rising global cancer burden and the lack of high-quality prospective data on CE in this population, CE in solid organ cancer patients represents a growing clinical challenge. Yet many aspects of this entity remain incompletely characterized. This review synthesizes the available literature on CE in solid tumor patients, including epidemiology, risk factors, clinical presentation, pathogenesis, diagnostic approaches, and management strategies. By highlighting the current evidence and knowledge gaps, we aim to provide clinicians and researchers with an updated framework for recognizing, diagnosing, and managing Candida esophagitis in this vulnerable and expanding patient population. We also highlight areas where research is needed, as well as future directions to guide researchers on this important yet underrecognized and understudied condition.

## 3. Epidemiology and Risk Factors

### 3.1. Epidemiology

Candida esophagitis is among the most common causes of infectious esophagitis. Its incidence has evolved, with changing patterns of immunosuppression. In the general population undergoing endoscopy, CE has a prevalence of under 1%, but this rate rises substantially in immunocompromised groups. Over the past two decades, the burden of CE has shifted away from HIV toward other conditions. Widespread antiretroviral therapy has dramatically reduced CE in AIDS patients, while the prevalence among cancer patients (especially those with solid tumors) has increased [5,11]. Notably, many cases likely go undetected due to subclinical disease. Asymptomatic or minimally symptomatic candidal esophagitis, particularly in patients with chemotherapy-induced neuropathy, opioid use, or radiation-related sensory loss, may contribute to the underestimation of its true prevalence in the solid cancer organ population. High-quality prospective studies are, however, lacking.

Solid organ malignancies now account for most CE cases in tertiary centers. In a recent study of 323 cancer patients with esophageal candidiasis, 89% had solid tumors (most commonly esophageal carcinoma) [2]. By contrast, invasive disseminated candidiasis remains more common in hematologic malignancies with prolonged neutropenia [12]. The overall increase in the prevalence of solid organ malignancies, the increase in CE in this population, and the asymptomatic or minimally symptomatic nature of this infection in this group underscore the fact that solid tumor patients represent an important and less recognized at-risk population for Candida esophagitis in the current era.

### 3.2. Risk Factors

Multiple risk factors often coexist to predispose cancer patients to Candida esophagitis. Key risk factors identified in the literature include the following.

- Cancer-associated immunosuppression: Advanced solid tumors impose a chronic immunosuppressive state. The tumor burden, cachexia, and paraneoplastic phenomena impair local mucosal defenses and systemic immunity, facilitating Candida overgrowth.
- Cytotoxic chemotherapy and radiation: Chemotherapy induces mucositis and disrupts the epithelial barrier, while thoracic radiotherapy causes esophagitis and edema. These mucosal injuries enable fungal adhesion and invasion. Case reports link severe, necrotizing Candida esophagitis to recent chest irradiation [13].
- Corticosteroid therapy: Corticosteroids (used for cancer-related edema, nausea, or immunotherapy toxicity) suppress neutrophil function and cell-mediated immunity [6].
- Broad-spectrum antibiotics: Antibiotic exposure alters the normal bacterial flora that competes with Candida, often precipitating fungal overgrowth.
- Acid suppression (PPIs/H2 blockers): Gastric acid is a natural defense against ingested pathogens. Acid-suppressive therapy, particularly PPIs, is consistently linked to higher rates of esophageal candidiasis. This warrants judicious prescribing, especially in patients receiving chemotherapy [5].
- Diabetes mellitus: Poorly controlled diabetes and hyperglycemia promote Candida adhesion to the esophageal mucosa and impair neutrophil function. Diabetes is frequently observed in CE patients (including those with cancer) and contributes to susceptibility [11].
- Malnutrition and weight loss: Nutritional compromise is both a consequence and cause of Candida infection. Cancer-associated malnutrition (cachexia, vitamin deficiencies) weakens mucosal barrier integrity and immune responses. Inadequate oral intake due to tumor- or treatment-related symptoms further exacerbates vulnerability to fungal invasion.
- Additional factors: Other comorbidities reported in CE patients include chronic esophageal cancer, an altered upper gastrointestinal tract anatomy, smoking, liver disease, alcoholism, and prior gastrointestinal surgery [14].

The cumulative effect of multiple risk factors is especially important: for instance, a malnourished patient on antibiotics, steroids, and a PPI is extremely susceptible to esophageal candidiasis.

It is noteworthy that the risk factors for CE differ in emphasis between solid tumor and hematologic malignancy patients. In hematologic cancers, profound neutropenia and lymphocyte dysfunction predispose patients to invasive candidiasis, often prompting antifungal prophylaxis. In solid tumor patients, localized mucosal disruption and pharmacologic factors dominate, and routine antifungal prophylaxis is not generally indicated in the absence of other risk factors. Recognition of these risk factors has practical implications. Clinicians should maintain a high index of suspicion for CE in cancer patients with the above exposures or comorbidities, even if classical symptoms are absent. Where possible, minimizing unnecessary antibiotic, corticosteroid, and PPI use in immunosuppressed patients may decrease the risk. However, routine prophylaxis in solid tumor patients is not supported by current evidence.

## 4. Clinical Manifestations

The clinical presentation of Candida esophagitis in solid tumor patients is variable, ranging from the typical esophageal symptoms to subtle or no symptoms at all. Odynophagia (pain on swallowing) and dysphagia (difficulty swallowing) are the hallmark symptoms. In some studies, odynophagia was reported in roughly 50% of CE cases and dysphagia in 25–50%. However, their frequencies vary widely based on the series. Retrosternal chest pain or epigastric burning is also described in 20–40% of cases, often exacerbated by swallowing. These symptoms can mimic reflux disease or radiation esophagitis, making clinical differentiation challenging without endoscopy. Non-specific upper gastrointestinal complaints—nausea, bloating, belching, or heartburn—occur in 10–20% and may lead to empiric treatment for gastritis or GERD, further delaying diagnosis or possibly worsening it given that PPIs are a risk factor for CE. Importantly, a significant subset of patients have atypical or minimal symptoms. Several studies emphasize that CE can be entirely asymptomatic in up to one third of cases, particularly in cancer patients with blunted pain perception from neuropathy or opioid use. For example, Samonis et al. documented that only 10 of 22 cancer patients with endoscopically proven CE reported even mild esophageal symptoms [15]. Similarly, a recent large series at a cancer center found that 33% of patients with esophageal candidiasis were asymptomatic [2]. This silent presentation is especially likely in those with diabetic or chemotherapy-induced neuropathy or with post-radiation sensory changes that diminish esophageal pain. Clinicians must therefore be vigilant for CE in high-risk patients, even in the absence of classic symptoms.

There is a poor correlation between symptom severity and disease severity on endoscopy or biopsies. Some patients with only mild discomfort harbor extensive esophageal invasion, whereas others with severe odynophagia have only modest endoscopic changes. In one report, patients with grade 3 (severe) candidal esophagitis were occasionally minimally symptomatic, while, conversely, grade 1 disease sometimes caused significant pain. Oropharyngeal candidiasis (thrush) is neither sufficiently sensitive nor specific in diagnosing CE: while thrush with esophageal symptoms likely indicates the presence of Candida esophagitis and empiric treatment is often recommended, the absence of thrush does not exclude the presence of CE [2,15].

Beyond acute symptoms, CE can have broader impacts on patient well-being. Painful swallowing often leads to reduced oral intake and malnutrition, which in turn can compromise tolerance to chemotherapy or radiation. Patients may develop an aversion to eating, exacerbating cachexia and dehydration. This can necessitate interruptions to cancer treatment or the provision of enteral/parenteral nutrition support. Psychologically, the addition of debilitating esophageal symptoms to an already burdened cancer patient can significantly erode their quality of life. Therefore, prompt antifungal therapy for CE often provides rapid relief. Studies note symptomatic improvements typically within a few days of treatment initiation, allowing patients to resume normal eating and continue cancer therapy with minimal delays [16].

In severe or untreated cases, local fungal proliferation can lead to serious complications, such as deep ulceration, bleeding, or, rarely, perforation [17]. Overall, the clinical spectrum of CE in solid tumor patients is broad, and maintaining a high index of suspicion is key to preventing the insidious decline associated with missed diagnoses.

## 5. Pathogenesis and Microbial Ecology

### 5.1. Fungal Pathogenesis in the Esophagus

*C. albicans* and other Candida species typically exist as harmless commensals in the human oropharyngeal and gastrointestinal microbiota. Transition to pathogenicity occurs when host defenses are compromised. In immunosuppressed cancer patients, multiple overlapping mechanisms contribute to this shift. Mucosal damage from chemotherapy or radiotherapy provides a foothold for Candida to adhere and invade. Immune dysfunction from corticosteroids or myelosuppressive therapy reduces the innate and adaptive responses that normally keep Candida in check. Disruption of normal flora by antibiotics and an increased gastric pH due to PPIs further promote Candida overgrowth.

Once Candida organisms attach to the esophageal epithelium, they employ several virulent strategies to establish infection [18].

- Adhesion and morphogenesis: *C. albicans* can transition from yeast form to filamentous hyphae and pseudohyphae. Both forms express adhesin proteins that mediate tight attachment to epithelial cells. Hyphal elongation enables the fungus to penetrate intercellular spaces, evading phagocytosis and causing cellular damage.
- Hydrolytic enzyme secretion: Candida secretes proteases, phospholipases, and other enzymes that digest host cell membranes and proteins, facilitating tissue invasion and nutrient acquisition. These enzymes contribute to cellular and mucosal damage and ulceration.
- Biofilm formation: Candida can form biofilms—complex communities encased in an extracellular matrix. Biofilms increase with cellular spread and aid in resistance to antifungal drugs and host defenses. This is relevant in patients with esophageal stents or feeding tubes and may underlie recurrent infections.

The result of these virulence factors is the characteristic pseudomembranous inflammation of Candidal esophagitis, i.e., white plaques composed of Candida mycelia, desquamated epithelial cells, and fibrin, with underlying mucosal erosion. In advanced cases, transmural invasion can occur, leading to necrosis.

### 5.2. Host Defense and Cancer-Related Impairments

Innate immunity (especially neutrophils and macrophages) provides a first line of defense by phagocytosing Candida organisms that breach the epithelium. Effective immunity against Candida in the esophagus primarily depends on cell-mediated mechanisms.

In patients with solid organ cancers, several factors undermine these defenses.

- Chemotherapy-induced leukopenia: While neutropenia in solid tumor regimens is typically less profound and prolonged than in leukemia or transplant conditioning, repeated cycles of chemotherapy can cause cumulative lymphocyte and neutrophil dysfunction.
- Radiation effects: Radiation to the chest or upper abdomen not only damages the mucosa but also alters local immune cell populations and cytokine signaling in the esophagus.
- Corticosteroids and immunomodulators: Steroids (and certain targeted cancer therapies) can broadly dampen both innate and adaptive immunity.
- Nutritional deficiencies: Deficiencies in micronutrients such as zinc, iron, and vitamins A, C, and D can impair epithelial integrity and immune cell function. Cancer patients with malnutrition often have multiple vitamin deficiencies that could contribute to reduced antifungal defenses.
- Anatomical factors: Factors such as esophageal obstruction or an altered anatomy can cause local environmental changes such as stasis that favor candidal growth.

Through these mechanisms, cancer therapies and the disease state itself create a permissive environment for Candida, tipping the balance from commensalism to pathogenic infection.

### 5.3. Mycology of Candida Esophagitis

*Candida albicans* remains the predominant species isolated in esophageal candidiasis, but there is increasing recognition of non-*albicans* Candida (NAC) species, especially in patients with prior azole exposure. Notable species include *Candida glabrata*, *C. tropicalis*, *C. krusei*, *C. lusitaniae*, and *C. parapsilosis* [2,19,20,21].

Each species has unique antifungal susceptibility patterns.

- *C. albicans*: Usually susceptible to fluconazole, although resistance can emerge. It continues to account for most cases.
- *C. glabrata*: Often exhibits reduced susceptibility or dose-dependent resistance to azoles. Treatment may require higher-dose fluconazole or an echinocandin. Recent years have seen more *C. glabrata* CE, likely related to heavy fluconazole prophylaxis in some settings [22].
- *C. krusei*: More likely to be resistant to fluconazole. Although a less common cause of esophagitis, its presence mandates the use of alternatives (e.g., voriconazole or echinocandins).
- *C. tropicalis* and *C. parapsilosis*: *C. parapsilosis* tends to be fluconazole-susceptible, while *C. tropicalis* can occasionally be resistant. Both are usually sensitive to echinocandins and voriconazole.

Antifungal resistance is a growing concern. Fluconazole-resistant *C. albicans* remains uncommon, but isolated cases are documented and typically involve prior azole exposure. Non-*albicans* species (especially *C. glabrata* and *C. krusei*) exhibit higher rates of resistance, including emerging echinocandin resistance in *C. glabrata*.

## 6. Diagnostic Approaches

The accurate diagnosis of Candida esophagitis in patients with cancer is essential to initiate appropriate therapy and prevent complications. However, diagnosis can be challenging because symptoms are non-specific and some cancer patients are completely asymptomatic. A combination of clinical vigilance and the judicious use of diagnostic tests is required.

Clinical suspicion: The diagnostic process begins with identifying at-risk patients and recognizing potential symptoms such as odynophagia or dysphagia; however, non-specific symptoms such as nausea, epigastric discomfort, and anorexia in solid organ cancer patients with a lack of clear reason should trigger suspicion for CE. The absence of thrush or the presence of alternative explanations (e.g., GERD) should not immediately rule it out. In those with oropharyngeal candidiasis, one should assume esophageal involvement until proven otherwise, given the strong association. Conversely, clinicians should remember that Candida esophagitis can occur without oral thrush in a substantial number of cases. Due to these nuances, a high index of suspicion must be maintained.

Endoscopy (EGD): Upper endoscopy with the visualization of the esophagus is the gold-standard diagnostic test for CE. On endoscopy, Candida infection classically appears as multiple white, adherent (difficult to wash) plaques or pseudomembranes on the esophageal mucosa. These plaques may be small and isolated (Kodsi grade I) or diffuse, coalescent with friability and ulceration (grade III–IV). An erythematous or edematous mucosa with white patches is strongly suggestive of candidiasis. Endoscopy not only permits the direct recognition of these lesions but also allows a biopsy or brush cytology to confirm the diagnosis. It is important to note that endoscopic findings alone are not pathognomonic—conditions like confluent HSV or CMV esophagitis, or even pill esophagitis, can sometimes create pseudo-plaque appearances. Thus, obtaining tissue or brushings for microscopic examination is advised, particularly in atypical or refractory cases.

Histopathology and cytology: Histologic confirmation remains the definitive diagnostic criterion for CE. Biopsy specimens, when stained with hematoxylin–eosin, periodic acid–Schiff (PAS), or Gomori methenamine silver (GMS), will demonstrate yeast and pseudohyphal forms invading the squamous mucosa. In many cases, no invasion or only superficial invasion is present. Because solid organ cancer patients have decreased host defenses, the presence of Candida should always be considered pathogenic, even if no invasion is seen on histology. The use of brush cytology, obtained by brushing the plaque and smearing on slides, is a less invasive alternative that can reveal yeast and hyphal elements with high sensitivity. Combining biopsy and brushings maximizes the diagnostic yield. Histopathology can also help to exclude or identify coexistent pathologies (e.g., viral inclusions of CMV). A distinctive histologic feature reported in Candida esophagitis is a band-like intraepithelial neutrophilic infiltrate with clusters of yeasts, which can help to differentiate it from other causes of esophagitis.

Microbiological culture: Although not always performed for an initial diagnosis, a culture of esophageal brushings or biopsy material is useful for species identification and antifungal susceptibility testing. This becomes particularly important in cases of refractory or recurrent esophagitis, where infection with an azole-resistant species (e.g., *C. glabrata* or *C. krusei*) is suspected. Routine culture is not necessary for straightforward cases that respond to therapy, but its role in guiding therapy for atypical or persistent cases is invaluable.

Radiographic studies: A barium swallow (esophagram) can show abnormalities in Candida esophagitis, although it is far less sensitive than endoscopy. Typical findings in double-contrast barium studies include irregular plaque-like filling defects or a ‘shaggy’ esophagus caused by barium coating the raised fungal plaques. Small, discrete ulcerations or nodularity might be seen in advanced cases. Strictures or decreased distensibility can occur in chronic cases due to scarring. While a suggestive radiographic finding in a high-risk patient could support a presumptive diagnosis, barium studies cannot definitively diagnose CE and may miss mild disease. They are generally reserved for patients who cannot undergo endoscopy due to severe thrombocytopenia or other contraindications.

Emerging diagnostics: Research is ongoing into non-invasive or adjunctive diagnostic tools for Candida infections. Serum β-D-glucan, a cell wall component of Candida, is a serum biomarker that is widely used for invasive candidiasis. However, its role in isolated esophageal candidiasis is unclear and likely limited, as the infection is usually superficial (β-D-glucan may be normal in localized disease). PCR-based assays for Candida in blood or tissue are being developed and could potentially aid in earlier detection, but distinguishing colonization from true infection is a challenge. Novel techniques like confocal laser endomicroscopy have been piloted to identify fungal elements in vivo during endoscopy, but these remain investigational. For now, direct visualization and histologic/cytologic confirmation remain the cornerstones [23,24].

Differential diagnosis: When evaluating esophageal white plaques or ulcerations, other causes must be considered. Viral esophagitides, particularly Herpes simplex virus (HSV) and Cytomegalovirus (CMV), can occur in immunosuppressed patients. HSV classically causes small, deep ulcers with a volcano-like appearance, while CMV causes larger linear ulcers; both usually lack diffuse white plaques, although co-infection with Candida can occur. Eosinophilic esophagitis can produce rings, plaques, or exudates that mimic candidiasis on gross appearance, but histology will show eosinophils rather than fungi. Pill esophagitis from medications (e.g., doxycycline, bisphosphonates) can cause localized ulcers and debris. Differentiating these conditions relies on a combination of biopsy (to look for viral inclusions or eosinophils) and culture/PCR as needed. In practice, if endoscopic findings suggest Candida, clinicians often initiate empiric antifungal therapy while awaiting pathology to exclude other diagnoses.

In summary, early endoscopy with biopsy is recommended for the definitive diagnosis of Candida esophagitis in cancer patients, especially given the possibility of asymptomatic disease and the need to exclude alternative etiologies. When endoscopy is not immediately available, a trial of fluconazole is sometimes used diagnostically (symptomatic improvement supports the diagnosis), but this approach risks masking other diagnoses and should be used cautiously.

## 7. Treatment Strategies

The management of Candida esophagitis in patients with solid organ cancers must be individualized, considering the severity of infection, the Candida species involved, drug interactions with chemotherapy, and the patient’s immune status and organ function. Key principles include the prompt initiation of effective systemic antifungal therapy and addressing modifiable predisposing factors (when possible) to prevent recurrence. In our opinion, in the absence of studies showing otherwise, CE should be treated in solid organ cancer patients when identified, even if the patient is asymptomatic, as untreated infections can have severe consequences and can contribute to nutritional decline.

### Antifungal Therapy

First Line—Fluconazole: Guidelines from the Infectious Diseases Society of America (IDSA) recommend systemic antifungal therapy for all cases of esophageal candidiasis, with oral fluconazole (200–400 mg daily for 14–21 days) as the first-line treatment. Fluconazole is often well tolerated and highly effective against *C. albicans* and most Candida species, achieving excellent tissue penetration in the esophagus. Fluconazole interacts with 14-demethylase, a cytochrome P-450 enzyme responsible for catalyzing the formation of ergosterol, a critical part of the fungal cell membrane. By inhibiting ergosterol synthesis, it increases cellular permeability and causes cellular damage. Fluconazole produces rapid symptom relief and high endoscopic cure rates. Fluconazole’s advantages include its oral formulation (important for outpatient therapy), favorable safety profile, and low cost. It has a higher response rate than amphotericin and lower relapse rates than echinocandins. The typical dose is 200 mg daily, which can be increased to 400 mg daily for extensive disease or if a non-*albicans* strain with dose-dependent susceptibility is suspected. An initial IV loading dose (e.g., 400–800 mg) can be given if rapid action is needed or if oral intake is poor. A clinical response is usually seen within 3–7 days; patients often report less pain and improved swallowing after just a few doses [25]. Specifically in solid organ cancer patients, it appears that treatment failure might be higher than in the general population, especially in people with esophageal cancer or obstruction [7].

Azole alternatives: Itraconazole, voriconazole, and posaconazole are other azole antifungals that have activity against Candida. They have a similar mechanism of action to fluconazole. Itraconazole oral solution (200 mg daily) is used for fluconazole-refractory mucosal candidiasis and has been shown to be effective 80% of the time for these patients. Voriconazole 200 mg (3 mg/kg) twice daily, either intravenous or oral, has broad-spectrum activity (including *C. krusei*); it can be considered for Candida esophagitis due to fluconazole-resistant species or in patients intolerant to fluconazole. However, voriconazole’s side effect profile (hepatic toxicity, visual disturbances) and metabolic interactions (especially with certain chemotherapeutic agents) require careful monitoring. Azole antifungals, except fluconazole, are substrates of CYP enzymes and drug transporters. They are particularly problematic in clinical practice because they inhibit multiple CYP isoenzymes and P-gp, cause multiple drug–drug interactions, and require therapeutic drug monitoring or careful dose adjustments. Interactions to note in solid organ cancer patients include opioids, oral anticoagulants, and certain chemotherapeutic agents such as vincristine, where they can increase the risk of neuropathy. They also interact with tyrosine kinase inhibitors [26]. Specifically, the co-administration of voriconazole with tyrosine kinase inhibitors metabolized by CYP3A4 (such as bosutinib, dasatinib, nilotinib, ibrutinib, and ponatinib) is likely to increase plasma concentrations of the tyrosine kinase inhibitor and the risk of adverse effects. If used concomitantly, dose reduction of the tyrosine kinase inhibitor and close clinical monitoring are recommended [27].

Posaconazole is a newer, extended-spectrum azole that is highly effective in oropharyngeal and esophageal candidiasis, including cases refractory to fluconazole. Posaconazole oral suspension or delayed-release tablets can be used when infections involve *C. glabrata* or other less susceptible strains. Posaconazole is generally well tolerated, with mild gastrointestinal upset being the most common side effect. These azole alternatives are usually reserved for fluconazole-refractory cases or when resistance is documented [28].

Echinocandins: The echinocandin class (caspofungin, micafungin, anidulafungin) is fungicidal against *Candida*, including many fluconazole-resistant strains. Echinocandins bind the enzyme involved in the biosynthesis of β-(1,3)-d-glucan, which leads to structural abnormalities in the fungal cell wall and cell death. They must be given intravenously but have the advantage of minimal toxicity and few serious drug interactions. Echinocandins are not typically first-line for esophageal candidiasis in most patients because fluconazole is effective and oral and has a lower relapse rate, although most of the relevant studies were performed in HIV-positive patients. However, in patients who cannot tolerate oral medication or who have infections with known azole-resistant species (*C. glabrata*, *C. krusei*), an echinocandin is an excellent option. In practice, caspofungin 50 mg IV daily, micafungin 150 mg IV daily, or anidulafungin 100 mg IV daily can be used for 2–3 weeks for Candida esophagitis. These are often chosen in hospitalized patients who are NPO or in whom *C. glabrata* infection is suspected (e.g., prior fluconazole prophylaxis). Once the patient improves, finishing therapy with an oral fluconazole is recommended [25] given the higher relapse rate on echinocandin monotherapy [25,29].

Amphotericin B: The polyene amphotericin B is an older agent with broad antifungal activity. Amphotericin B acts by binding to ergosterol in the cell membrane, causing the formation of ion channels and resulting in depolarization and concentration-dependent cell killing. It is now reserved for refractory or severe cases of Candida esophagitis due to its toxicity profile and lower efficacy. Amphotericin B deoxycholate (conventional form) causes infusion reactions and nephrotoxicity, making it a less appealing choice if other options are available. Lipid formulations (liposomal amphotericin B or amphotericin B lipid complex) are better tolerated but very costly, so they are typically used only as salvage therapies [29].

Topical and adjunct therapies: Unlike oropharyngeal candidiasis (thrush), topical agents like nystatin suspension or clotrimazole troches are not adequate for esophageal candidiasis. These agents do not achieve therapeutic levels in the distal esophagus and should not be used as the sole therapy for CE. They may be given concurrently to treat oral thrush, but systemic therapy is always required for esophageal involvement. There has been interest in local prophylactic measures in high-risk situations. A study using amphotericin B lozenges (not absorbed) during radiation therapy to the chest showed reductions in radiation esophagitis severity.

Treatment duration and follow-up: The recommended treatment length for Candida esophagitis is 14–21 days, depending on the clinical response and the patient’s immune reconstitution. Longer courses may be necessary for patients who remain immunosuppressed or who have persistent infection. Clinical improvement is expected within a week; a lack of improvement by day 5–7 of therapy should prompt re-evaluation (repeat endoscopy to obtain cultures or check for alternative diagnoses). Repeat endoscopy to document healing is generally not required if symptoms resolve but should be considered if the patient has ongoing symptoms or risk factors for esophageal stricture. Throughout therapy, monitoring for antifungal side effects is important: azoles can cause hepatotoxicity and QT interval prolongation, and they interact with many chemotherapeutic and supportive care drugs (e.g., vincristine, certain TKIs, warfarin, direct-acting oral anticoagulants). Echinocandins have minimal drug interactions but can cause mild liver enzyme elevations, so periodic labs are reasonable in longer courses. Coordination with the oncology team is crucial to adjust treatments if needed—for instance, certain chemotherapy agents metabolized by CYP3A4 might need dose changes if voriconazole is used.

## 8. Refractory and Recurrent Disease

Most cases of Candida esophagitis respond to initial therapy. However, in a subset of patients, the infection is refractory (persistent despite appropriate treatment) or recurrent (initial response followed by relapse shortly after therapy). A subset of cancer patients with CE experience refractory or relapsing disease. Management in these situations includes the following.

- Reassess and identify species/resistance: If not already done, perform endoscopy with a biopsy and culture of the esophageal lesions. It is critical to determine if a resistant Candida species (e.g., *C. glabrata* with elevated fluconazole MIC or *C. krusei*) is present, as well as excluding alternative diagnoses or co-infections (such as superimposed herpes or CMV esophagitis). Underlying esophageal cancer is an independent predictor of fluconazole failure, meaning that local tumor effects may sometimes impede clearance [2]. In such cases, more aggressive or prolonged therapy might be needed.
- Optimize antifungal regimen: For refractory CE, options include higher-dose fluconazole (e.g., 400–800 mg daily, if strain is susceptible), switching to a different azole (voriconazole or posaconazole), or using an echinocandin or amphotericin B as salvage. For example, a fluconazole-refractory *C. glabrata* infection would warrant an echinocandin (caspofungin/micafungin) for at least 2–3 weeks. If there is doubt about susceptibility, therapy should be tailored to culture results. Combining antifungals is generally not necessary for esophageal candidiasis and increases toxicity.
- Secondary prophylaxis: In patients with multiple relapses of CE (often those with ongoing immunosuppression, such as continued chemotherapy or corticosteroids), a suppressive strategy can be considered. This might involve fluconazole 100–200 mg thrice weekly or daily for a defined period to prevent recurrence. The IDSA guidelines do not routinely recommend chronic suppressive therapy for mucosal candidiasis due to concerns about resistance and cost, but, in select cases (e.g., a patient who has had two or more relapses, interfering with cancer care), the benefits may outweigh the risks. Such prophylaxis should be re-evaluated periodically.
- Address predisposing factors: Ensure that any removable risk factors are managed; for instance, reduce or discontinue unnecessary PPI use, taper steroids if feasible, improve nutritional support, and treat co-existing oropharyngeal candidiasis fully. In some cases, controlling the underlying cancer or immunosuppressive state is the ultimate solution to break the cycle of recurrence.

Even with aggressive management, some immunocompromised patients will have chronic, intermittent Candida esophagitis. The goal is to minimize the symptom burden and prevent serious complications. Refractory CE due to multi-drug-resistant Candida (e.g., *C. glabrata* resistant to both azoles and echinocandins) is fortunately very rare, but it may require innovative approaches such as the combination antifungals or investigational agents (e.g., *iborafungerp* or *oteseconazole* in development for Candida infections).

## 9. Complications and Prognosis

When recognized and treated promptly, Candida esophagitis generally carries a favorable prognosis, with rapid symptom improvements and mucosal healing. It is unusual for CE itself to be a direct cause of mortality. However, in the context of cancer, CE can significantly worsen patient outcomes indirectly through malnutrition, interruptions of therapy, or precipitating other complications.

Local complications of severe or untreated Candida esophagitis include the following.

- Necrotizing esophagitis: Deep fungal invasion can rarely lead to transmural necrosis of the esophagus. This was reported in particular in patients who received concurrent thoracic radiation, where mucosal injury was already present. Necrotizing Candida esophagitis may present with severe chest pain, odynophagia, and signs of systemic infection. CT imaging may show esophageal wall thickening or even air if perforation is impending. This condition requires urgent antifungal therapy and often surgical consultation.
- Esophageal perforation and mediastinitis: Progression of necrosis can result in a full-thickness perforation of the esophagus, spilling the contents into the mediastinum. Fungal mediastinitis or an abscess can ensue, which are life-threatening. Case reports of Candida esophageal perforation describe high mortality despite aggressive surgical and medical management. Fortunately, this is extremely rare in current practice, largely preventable by the early treatment of CE.
- Hemorrhage: Fungal ulcers can erode into esophageal blood vessels, causing bleeding. There are older reports of patients with CE presenting with hematemesis or melena due to ulceration into submucosal arteries. The risk may be heightened in patients with thrombocytopenia or those on anticoagulation. Endoscopic therapy (e.g., hemostatic clipping) along with antifungals is used if bleeding occurs.
- Stricture formation: Chronic Candida infection can lead to fibrosis and esophageal stricture after healing. This manifests as persistent dysphagia even after the infection is cleared and requires dilation procedures for management. In the HIV era, candidal strictures were occasionally seen in patients with late diagnoses; in cancer patients, one might see this if the diagnosis of CE was delayed or in those with recurrent infections causing repeated injury.
- Aspiration pneumonia: Painful swallowing might lead patients to aspirate liquids or secretions. Additionally, if CE causes regurgitation, there is a risk of aspirating Candida-colonized material into the lungs, potentially contributing to fungal pneumonia in a debilitated host. This is a secondary complication to be mindful of in bedbound or neurologically impaired patients.

Beyond these direct complications, the indirect impacts of CE are highly significant in oncology care. Perhaps the most important is nutritional deterioration: odynophagia and dysphagia often force patients to restrict their diets, resulting in weight loss and dehydration. This nutritional decline can reduce the tolerance to chemotherapy (leading to dose reductions or delays) and impair wound healing or performance status. In some cases, active cancer treatment must be postponed until esophagitis is brought under control. Each delay in cancer therapy could affect disease outcomes, especially in curative settings. Thus, preventing and promptly treating CE is crucial in maintaining the continuity of cancer treatment. Occasionally, nausea, anorexia, or weight loss are the symptoms. These are often difficult to distinguish from opioid adverse events or cancer cachexia except with an upper endoscopy. The threshold for an upper endoscopy in solid organ cancer patients should be very low, as this can have a significant impact on the overall quality of life and prognosis of the patient and is simple to reverse.

Quality of life is another consideration. Patients with cancer already face significant physical and psychological stress; superimposed esophageal pain and difficulty eating add further burdens. The effective treatment of CE can markedly improve comfort and allow the resumption of normal oral intake, thereby improving patients’ daily functioning and morale.

In terms of overall prognosis, Candida esophagitis in solid tumor patients is typically a manageable condition with low attributable mortality. However, for the majority of solid tumor patients, Candida esophagitis is a transient and treatable condition when identified. The early use of fluconazole typically leads to a rapid improvement, and, with appropriate supportive care (nutritional support, pain control), the infection resolves without long-term sequelae. Clinicians should remain vigilant for CE in any high-risk patient to ensure that these generally good outcomes are achieved. Generally, it is necessary to be aware of Candida esophagitis before it causes irreversible complications. Table 1 summarizes the main antifungal treatment options and their outcomes in cancer patients with CE. Table 2 is a reference for pharmacokinetic and pharmacodynamic profile of common systemic antifungal agents and their relevant interactions with common systemic therapies used in patients with solid organ malignancies. 

## 10. Future Directions

Despite advances in understanding and managing Candida esophagitis, several knowledge gaps and unmet needs remain, particularly for patients with solid organ cancers. Most available studies are retrospective and involve heterogeneous patient populations, making it challenging to draw firm conclusions specific to solid tumors. Going forward, research and clinical efforts should focus on the following areas.

- Improved diagnostic tools: Symptom-based diagnosis is unreliable; as noted, a substantial proportion of patients are asymptomatic or present with non-specific complaints. While endoscopy with histopathology remains the gold standard, it is invasive and not always feasible in frail oncology patients. Non-invasive biomarkers (e.g., serum β-D-glucan, Candida-specific PCR on saliva or stool) need further exploration to aid early diagnosis and to distinguish mere colonization from active infection and identify local infection. Advanced imaging techniques or point-of-care tests that could detect Candida in the esophagus without full endoscopy would be valuable in high-risk cases.
- Antifungal resistance surveillance: The species distribution in CE is evolving, with the increasing isolation of azole-resistant non-*albicans* species such as *C. glabrata* and *C. krusei*. Oncology centers should implement routine surveillance of Candida isolates and their antifungal susceptibility. This could involve the periodic culture of candidal colonization (for example, oral or stool cultures in patients on prophylaxis) or at least the analysis of all clinical Candida isolates for resistance patterns. The early identification of resistant strains would enable tailored therapy and potentially curb the emergence of difficult-to-treat infections. Collaboration in reporting antifungal resistance data across institutions would help to update guidelines on empirical therapy.
- Targeted prophylaxis strategies: Universal antifungal prophylaxis is not recommended for solid tumor patients, as the overall incidence of invasive candidiasis is low and indiscriminate use can drive resistance. However, there may be subsets of patients who would benefit from prophylaxis or pre-emptive therapy. Candidates might include those receiving intense chemoradiation to the esophagus (where mucositis is expected) or patients on long-term high-dose corticosteroids. Small studies (e.g., using amphotericin lozenges during radiation) hint at possible benefits. Prospective randomized trials are needed to determine if targeted prophylaxis in such groups can reduce the incidence of CE without undue risk. The balance between prophylactic benefit and the risks of drug resistance, cost, and interactions must be carefully studied.
- Novel therapeutics and adjuncts: The antifungal armamentarium for mucosal candidiasis may expand in the coming years. New antifungal agents (such as *ibrexafungerp*, an oral glucan synthase inhibitor, or oteseconazole, a novel azole) could offer alternatives for azole-resistant cases or allow oral step-down therapy in strains currently needing IV drugs. Immunomodulatory therapies that boost mucosal immunity (for example, recombinant cytokines or probiotics to restore microbiota balance) are another intriguing area. Additionally, techniques like photodynamic therapy—in which a photosensitizer and endoscopic light are used to kill fungi—have shown promise in case reports on patients with concurrent esophageal cancer. While not a primary therapy, photodynamic therapy or topical antifungal gels might serve as adjuncts to systemic treatment in the future, especially for localized lesions. Ongoing research into Candida biofilm disruption and fungal vaccines may also eventually impact prevention and management strategies.
- Integration with oncologic care: Multidisciplinary management is essential to address the multifactorial issues in these patients. Nutritionists should be involved early when CE is diagnosed, to support caloric intake during acute treatment. Oncology teams may need to adjust chemotherapy schedules or provide growth factors to aid immune recovery to help resolve the infection. Palliative care can assist with pain management for odynophagia. Developing protocols within cancer centers for screening high-risk patients (for example, performing endoscopy in patients with head/neck or thoracic cancers before starting radiation or performing an empiric fluconazole trial in those with weight loss and risk factors) could be explored. Understanding the impact of CE on cancer outcomes (e.g., does it measurably affect survival or tumor control by causing treatment delays?) through prospective data would help to drive home the importance of prevention and early treatment among the oncology community.
- Artificial intelligence (AI): Artificial intelligence can play multiple roles in CE. For example, large data sets can be used to better understand risk factors and produce a model for identifying patient characteristics or treatments that place patients at high risk. In addition, deep learning and convolutional neural networks are being explored to identify Candida species from images of fungal stains, possibly allowing the early tailoring of treatments [30].

In summary, while Candida esophagitis in solid organ cancer patients is now on the clinical radar, much work remains to optimize its diagnosis, treatment, and prevention. Focused research to clarify its true prevalence, refine risk stratification, and develop innovative therapies will improve care for these patients. As the population of cancer survivors grows and therapy intensification continues, addressing these knowledge gaps is becoming increasingly important.

## 11. Conclusions

Candida esophagitis (CE) is a clinically significant opportunistic infection in patients with solid organ cancers, driven by multifactorial risks including chemotherapy, radiotherapy, corticosteroids, antibiotics, and acid-suppressive therapy. Unlike in hematologic malignancies, where profound neutropenia predisposes patients to disseminated fungal disease, CE in solid tumor patients is typically localized to the esophagus. Nonetheless, its impact on nutrition, quality of life, and the continuity of oncologic treatment is substantial.

A high index of suspicion is required to diagnose CE promptly, given the often non-specific or silent presentation. Endoscopic evaluation with histopathologic confirmation remains the diagnostic gold standard. Adjunctive, less invasive diagnostics are needed to support early detection in those who cannot undergo immediate endoscopy. The first-line therapy is fluconazole, which yields high cure rates in *C. albicans* infection. Alternative azoles (itraconazole, voriconazole, posaconazole), echinocandins, or amphotericin B are reserved for resistant or refractory cases. The emergence of non-*albicans* species such as *C. glabrata* and *C. krusei* presents an ongoing challenge, underscoring the value of obtaining cultures in non-responding cases and tailoring therapy accordingly. Fortunately, with appropriate treatment, the prognosis of CE is generally favorable: symptoms improve within days and serious complications are rare if therapy is initiated early.

Recurrent or refractory disease does occur in a minority of patients, particularly those with persistent immunosuppression or esophageal cancer. Such cases demand a reassessment of the organism’s identity and susceptibilities, as well as the consideration of maintenance antifungal strategies. Preventing relapse also involves addressing underlying risk factors (for example, reducing unnecessary PPI or steroid use).

Looking ahead, because very few studies have been performed in the solid tumor population, prospective studies are needed to better define the true incidence and outcomes of CE in this population. Research into improved diagnostics (e.g., biomarker assays), antifungal stewardship (to limit resistance development), and selective prophylaxis in high-risk groups will help to refine management [31]. Novel therapies, including non-azole antifungals and adjunctive modalities, warrant further exploration in clinical trials. A multidisciplinary approach involving oncologists, gastroenterologists, infectious disease specialists, pharmacists, and nutrition support is essential to mitigate the burden of CE and ensure that patients can continue their cancer treatments with minimal interruption. Furthermore, while cytotoxic systemic therapy and some local cancer therapies, such as radiation, are known to increase the risk of CE, very little is known or written about the effects of immune checkpoint inhibitors on CE, and prospective studies should take this into consideration [32].

In summary, Candida esophagitis in solid organ cancer patients is an under-recognized but important complication of modern cancer therapy. By maintaining vigilance for this condition, promptly confirming the diagnosis, and implementing effective antifungal treatment, clinicians can markedly improve patients’ comfort and nutritional status, thereby optimizing the overall care and outcomes of patients with cancer.

## Figures and Tables

**Table 1:** Antifungal treatment options for Candida esophagitis in cancer patients.

Therapy (Route)
Fluconazole (oral or IV)
Itraconazole (oral)
Voriconazole (oral or IV)
Posaconazole (oral)
Echinocandins (IV)—e.g., caspofungin, micafungin, anidulafungin
Amphotericin B deoxycholate (IV)
Nystatin suspension (oral), clotrimazole troches

**Table 2:** Detailed pharmacokinetic and pharmacodynamic profile of common systemic antifungal agents (triazole azoles, echinocandins, and amphotericin B) and considerations in patients receiving chemotherapy or targeted anticancer agents.

Agent (Class)	Bioavailability and Route	Half-Life (t1/2)	Metabolism and Excretion	Mechanism of Action	Major Adverse Effects	Key Drug–Drug Interactions (Oncology Context)
Fluconazole (triazole azole)	Oral 90% bioavailable; available IV and PO. Food does not significantly affect absorption.	30 h (in normal renal function); prolonged in renal impairment (requires dose adjustment).	Minimal hepatic metabolism; ≥60% excreted unchanged in urine (renal elimination via glomerular filtration).	Inhibits fungal lanosterol 14α-demethylase, blocking ergosterol synthesis in cell membranes—fungistatic against most Candida/yeasts.	Generally well tolerated; may cause hepatotoxicity and QT interval prolongation (arrhythmia risk). GI upset and rash are occasional.	CYP inhibitor (moderate)—fluconazole strongly inhibits CYP2C9 and moderately CYP3A4, raising levels of many CYP3A4 substrates. Notably, co-administration of fluconazole can increase vincristine exposure, risking neuropathy and ileus. It also elevates levels of certain taxanes and kinase inhibitors metabolized by CYP3A4 (e.g., imatinib), so dose reduction or heightened monitoring of the anticancer drug is recommended. Concomitant use with the alkylator ifosfamide (a CYP3A4 substrate) has been reported to increase ifosfamide neuro- and nephrotoxicity. Rifampin (CYP inducer) can significantly reduce fluconazole levels.
Itraconazole (triazole azole)	Oral bioavailability 55% (capsule form; variable absorption)—acid and food enhance capsule absorption, whereas cyclodextrin oral solution has better bioavailability when taken on empty stomach. IV formulation available.	30 h at steady state. Reaches steady state in ~1–2 weeks without loading dose (due to long t1/2).	Extensive hepatic metabolism via CYP3A4 to active hydroxy-itraconazole. Highly lipophilic (large Vd ~11 L/kg; >99% protein-bound). Eliminated mostly as metabolites in bile/feces (~54%) and urine (~35%).	Inhibits fungal 14α-demethylase (ergosterol synthesis)—fungistatic broad-spectrum azole. Also inhibits fungal P-glycoprotein transporters, enhancing intracellular drug concentrations.	Hepatotoxicity, GI intolerance (nausea, diarrhea), and dose-related negative inotropy (avoid in heart failure; itraconazole can precipitate or worsen CHF). Can prolong QT interval (risk of arrhythmia).	Potent CYP3A4 inhibitor—causes numerous interactions. Itraconazole can dramatically increase levels of drugs metabolized by CYP3A4 (and also inhibits P-gp/BCRP transporters). In oncology, co-administration often leads to toxicity of vinca alkaloids (e.g., vincristine (rarely fatal) neuropathy/paralytic ileus) and taxanes (enhanced neutropenia or neurotoxicity). Strongly contraindicated with certain agents (e.g., some statins and QT-prolonging drugs). If used with CYP3A4-metabolized TKIs or EGFR inhibitors, dose adjustments and drug level monitoring are necessary. Due to its CYP3A4 and P-gp inhibition, itraconazole should generally be avoided with vinca alkaloids or reduced-dose vincristine used as clinically necessary.
Voriconazole (triazole azole)	Oral bioavailability 96% in healthy adults (absorption is independent of gastric pH). IV and oral (tablet, suspension) formulations available. Note: absorption may be lower in critically ill patients.	6 h (dose-dependent). Exhibits non-linear kinetics—half-life increases with higher doses/concentrations due to saturable metabolism. Steady state achieved after ~5–6 doses with standard regimens.	Primarily hepatic via CYP2C19 (major), 2C9, and 3A4. Voriconazole is a substrate and strong inhibitor of CYP2C19/2C9, moderate inhibitor of CYP3A4. Metabolism is saturable; small dose increases can cause large AUC increases. ~80% of dose recovered in urine as metabolites (only ~2% unchanged).	Inhibits fungal 14α-demethylase (ergosterol synthesis)—fungistatic against yeasts; fungicidal against some molds (Aspergillus). Also has a short post-antifungal effect in Aspergillus.	Hepatotoxicity (elevated LFTs) is common. Dose-related visual disturbances (reversible blurred vision, altered color perception) occur in up to 30% of patients shortly after doses. Hallucinations and encephalopathy can occur at high concentrations. Photosensitivity (sunlight-induced rash, cheilitis) is a notable long-term effect, and long-term use has been linked to cutaneous malignancies. Prolongs QT interval (arrhythmia risk).	Multiple CYP interactions. In oncology, co-administered vincristine or other vinca alkaloids can lead to severe neurotoxicity (recommend avoidance or chemo dose reduction). Voriconazole can also elevate levels (and toxicities) of TKIs (e.g., dasatinib, pazopanib), BCR-ABL inhibitors, and bortezomib; careful monitoring or alternative antifungal (e.g., an echinocandin) is advised.
Posaconazole (triazole azole)	Oral suspension has poor and variable absorption (requires high-fat meal or nutritional supplement to enhance uptake). Newer delayed-release tablet and IV formulations achieve much higher and more reliable absorption (tablet AUC three times that of suspension at equal doses), with less dependence on food or gastric pH.	25–35 h (long-acting). t1/2 ~20–30 h after a single dose, ~35 h at steady state with the DR tablet. Steady state reached in ~7–10 days (or sooner with loading dose).	Primarily hepatically metabolized by UGT1A4 glucuronidation (phase II); minimal CYP450 metabolism. P-glycoprotein substrate. Eliminated mostly in feces—~77% of dose in feces (66% as unchanged parent drug) and ~14% in urine (as glucuronide metabolites). Potent inhibitor of CYP3A4 (like other azoles).	Inhibits fungal 14α-demethylase (ergosterol synthesis)—broad-spectrum fungistatic activity (including Aspergillus and Mucorales).	Hepatic toxicity (elevated transaminases) occurs in a minority of patients. GI side effects (nausea, diarrhea) are relatively common. Notably causes QT prolongation at high concentrations so monitor electrolytes and ECG if combined with other QT-prolonging drugs.	Strong CYP3A4 inhibitor: posaconazole greatly increases levels of co-administered CYP3A4 substrates. This is clinically relevant for many chemotherapies—e.g., vincristine neurotoxicity can be severe when posaconazole is used for prophylaxis (azoles should be avoided during vinca alkaloid therapy). Levels of certain tyrosine kinase inhibitors (e.g., ibrutinib, vemurafenib) and bortezomib are elevated by posaconazole, increasing toxicity (monitor drug levels or avoid combination). Posaconazole’s inhibition of 3A4 is so potent that alternative antifungals (echinocandins or isavuconazole) are often preferred in patients on intensive chemotherapy to minimize interactions. Acid-suppressing drugs and mucositis can reduce absorption of the suspension, so the tablet/IV forms are used in such patients.
Caspofungin (echinocandin)	No oral absorption (IV only).	9–11 h effective half-life (β-phase); terminal t1/2~27 h. Once-daily dosing is used (some accumulation ~50% in first week).	Hepatic non-CYP metabolism: peptide hydrolysis and N-acetylation to inactive metabolites. No significant CYP involvement. ~41% of dose recovered in urine, ~34% in feces as metabolites. Moderate (~95%) protein binding.	Inhibits β-(1,3)-D-glucan synthase, preventing β-glucan formation in the fungal cell wall—fungicidal against most Candida species, fungistatic against Aspergillus. Disrupts cell wall integrity, leading to cell lysis.	Well tolerated. Histamine-mediated infusion reactions (transient rash, flushing, pruritus) can occur if infused too quickly. Mild hepatic toxicity noted infrequently (transaminase elevations); risk may increase when combined with cyclosporine. Rarely, can cause fever, headache, or phlebitis at injection site. No significant renal toxicity.	Minimal drug interactions: caspofungin does not inhibit CYP450 enzymes and has low potential for PK interactions. It is often the antifungal of choice in patients on complex chemotherapy because it will not raise levels of drugs like vincristine or taxanes. Only minor interactions observed: co-administered cyclosporine increased caspofungin AUC by ~35% (monitor LFTs).
Amphotericin B (polyene)	Negligible oral absorption; must be given IV for systemic use. Available as conventional deoxycholate formulation and lipid formulations (e.g., liposomal amphotericin B).	Biphasic half-life: 15–24 h (plasma t1/2 β-phase) for conventional amphotericin B. Terminal elimination is prolonged (drug persists in tissues for weeks; liposomal form t1/2~≥2 days).	No significant metabolism—amphotericin B is eliminated unchanged. Distributed extensively into tissues (sequesters in liver and spleen). Slowly excreted via both biliary and renal routes: 40% of dose recovered in feces and 20% in urine within 7 days. Highly protein-bound (95–99%).	Binds ergosterol in fungal cell membranes, forming transmembrane pores that increase permeability. This leads to leakage of cell contents (K^+^, Mg^2+^) and cell death. Fungicidal against a broad range of fungi (yeasts and molds). Also causes oxidative damage to fungal cells.	Infusion-related reactions in ~50% (fever, chills, rigors, hypotension)—premedication (antipyretics, antihistamines) often used. Nephrotoxicity is dose-limiting: occurs in majority of patients on conventional formulation (renal vasoconstriction and tubular damage cause rising creatinine, electrolyte wasting).	No CYP interactions (amphotericin is not metabolized). Pharmacodynamic interactions are significant: concurrent use of other nephrotoxic agents should be avoided. If possible, suspend or dose-reduce nephrotoxic chemotherapy (e.g., platinum compounds) during amphotericin B therapy, or use liposomal amphotericin to mitigate renal risk.

## Data Availability

All data is contained within this article.

## Abbreviations

AIDS	Acquired immunodeficiency syndrome
CE	Candida esophagitis
CMV	Cytomegalovirus
CT	Computed tomography
CYP	Cytochrome P450 (drug-metabolizing enzyme system)
CYP2C19/CYP2C9/CYP3A4	Specific cytochrome P450 enzyme isoforms
DR (tablets)	Delayed-release (tablets)
EGD	Esophagogastroduodenoscopy (upper endoscopy)
GI	Gastrointestinal
GMS	Gomori methenamine silver (stain)
H&E	Hematoxylin and eosin (stain)
H2 blockers	Histamine-2 receptor blockers (acid-suppressing drugs)
HIV	Human immunodeficiency virus
HSV	Herpes simplex virus
IDSA	Infectious Diseases Society of America
IV	Intravenous
kg	Kilogram(s)
MIC	Minimum inhibitory concentration
NAC	Non-*albicans* Candida
NPO	Nil per os (nothing by mouth)
PAS	Periodic acid–Schiff (stain)
PCR	Polymerase chain reaction
P-gp	P-glycoprotein (drug transporter)
PPI/PPIs	Proton pump inhibitor(s)
QT (interval)	QT interval on an ECG/EKG (cardiac electrical repolarization measure)
TKIs	Tyrosine kinase inhibitors
